# Rotating Focused Field Eddy-Current Sensing for Arbitrary Orientation Defects Detection in Carbon Steel

**DOI:** 10.3390/s20082345

**Published:** 2020-04-20

**Authors:** Zhiyuan Xu, Xiang Wang, Yiming Deng

**Affiliations:** 1 School of Mechanical Engineering, Xiangtan University, Xiangtan 411105, China; wangxiang_0107@163.com; 2 Department of Electrical and Computer Engineering, Michigan State University, East Lansing, MI 48824, USA; dengyimi@egr.msu.edu

**Keywords:** eddy current testing, rotating electromagnetic field, focusing coil, defect detection, signal-to-noise ratio, non-destructive testing

## Abstract

This paper presents a rotating focused field eddy-current (EC) sensing technique, which leverages the advantages of magnetic field focusing and rotating magnetic field, for arbitrary orientation defects detection. The sensor consists of four identical excitation coils orthogonally arranged in an upside-down pyramid configuration and a giant magneto-resistive (GMR) detection element. The four coils are connected to form two figure-8-shaped focusing sub-probes, which are fed by two identical harmonic currents with 90 degrees phase difference. A finite element model-based study of arbitrary orientation defects detection was performed to understand the probe operational characteristics and optimize its design parameters. Probe prototyping and experimental validation were also carried out on a carbon steel plate specimen with four prefabricated surface-breaking defects. In-situ spot inspection with the probe rotating above the defect and a manual line-scan inspection were both conducted. Results showed that the probe has the capability of detecting defects with any orientations while maintaining the same sensitivity and the defect depth can be quantitatively evaluated by using the signal amplitude. Compared with the existing rotating field probes, the presented probe does not require additional excitation adjustment or data fusion. Meanwhile, due to its focusing effect, it can generate a strong rotating magnetic field at the defect location with a weak background noise, thus yielding superior signal-to-noise ratio.

## 1. Introduction

Eddy current testing (ECT) has been widely applied in the detection of surface and subsurface defects in conductive materials. It utilizes a magnetic probe to generate an alternating magnetic field, which induces an eddy current in the specimen and measures the secondary magnetic field produced by the eddy current. Physical anomalies and discontinuities in material properties existing in the specimen can then be identified by analyzing the probe output signals [1]. The ECT probe plays roles as the actuating source and also the receiving sensor for damage detection. Throughout the literature, different ECT probe types have been reported and their operation modes are generally classified into four categories—absolute, differential, reflection and hybrid [1,2,3]. Reflection probes have separate excitation and detection sensors, which can be individually optimized for their intended applications [3,4]. In the past few decades, modern magnetic sensors such as Hall devices; anisotropic, giant and tunnel magneto resistive (AMR, GMR and TMR) sensors and superconducting quantum interference devices (SQUIDs) are becoming alternatives of detection coils due to their intrinsic advantages over induction coils, including their higher sensitivity at low frequency and smaller sensing footprint for small defects [5,6,7]. However, for the excitation purpose, a wire-wound coil, no matter air-cored, ferrite-cored or printed, is still the primary and most common choice.

As the ECT probe’s capability and accuracy for defect detection correlate with the noise level of eddy current signals, one main objective in the ECT probe design is to achieve an output with high signal-to-noise ratio (SNR) [8,9,10]. This can be achieved by improving the probe coil configurations and excitation modes, examples of which have been extensively reported in previous studies [2,10,11,12,13].

Following the notion of the figure-of-eight coil used in deep transcranial magnetic stimulation [14,15], a so-called “figure-8-shaped” focusing probe was introduced by Zhang et al. [16,17]. It consists of two coils tilted oppositely like a butterfly over the specimen. Compared with the circular pancake coil, it can induce unidirectional eddy currents with greater current density and focus them in a smaller region in the conductor. Their study also indicates that the eddy current density reaches its maximum beneath the probe center at which the eddy current produced by circular pancake probes tends to be zero. This type of probe shows great sensitivity to the defect perpendicular to the eddy current but would lose its detectability when defects orient in parallel along the eddy current direction [18].

To cope with the practical situation that the defect orientation is not known in advance or is unpredictable, two methods have been developed—rotationally scanning on the suspicious zone [19,20,21] and developing a rotating current excitation [22,23,24,25,26]. They have the same underlying physics for the sensing mechanism but the latter is superior to the former because it avoids the inevitable noise caused by mechanically rotating and is suitable for a C-scan or even a line scan. In general, this method employs two orthogonally placed coils driven by two current excitations with 90 degrees phase difference to result in a uniform and rotating eddy current (EC) field in the specimen. By doing this, the probe shows the same sensitivity to arbitrarily oriented defects. Yang et al. [22] proposed a printed-circuit-board (PCB)-based rotating field EC-GMR probe for the detection of embedded cracks in riveted multilayer structures. Each side of the PCB was fabricated with a planar coil, resulting in a structural lift-off deviation for the two excitation coils. Thus, a compensation method was required to form a circularly rotating field. This probe structure was then further developed by Ye et al. [23,24], including GMR arrays to eliminate the impact of background field and applying TMR to replace GMR for increasing the spatial resolution. In a similar way, Repelianto et al. [25] presented a rotating uniform eddy current probe with two pairs of orthogonally installed rectangular excitation coils and a small detection coil. Since the stacked excitation coils have quite different lift-off distances, they are well adjusted with different numbers of turns and excitation currents to make a rotating field. Recently, Wang et al. [26] presented a probe with a printed right-triangle excitation coil and two TMR arrays placed on the triangle’s two legs. In order to make an equivalent rotating field, acquired TMR data were fused by a series of complex algorithms including shifting, rotating and multifrequency mixing. In short, to generate a rotating field, additional processing like excitation adjustment or data fusion after acquiring the EC signals is required for state-of-the-art EC probe design.

This work proposed a novel rotating field EC probe by integrating the figure-8-shaped coil focusing scheme. Two pairs of focusing coils are orthogonally arranged at the same lift-off, which thus avoids additional excitation adjustment for generating a rotating EC field and also makes the field concentrated with high density. A prototype probe with a GMR sensor as the receiver was developed and its capacity for arbitrary orientation defects was investigated using finite element analysis and an experiment test. The GMR sensor was mounted at the probe center without increasing the probe’s design lift-off. The experimental signal had superior SNR and its amplitude can be used to evaluate the depth of arbitrarily oriented defect, demonstrating the effectiveness of the proposed probe.

## 2. Principle

### 2.1. Dual-Coil Focusing Scheme

**Figure 1:**
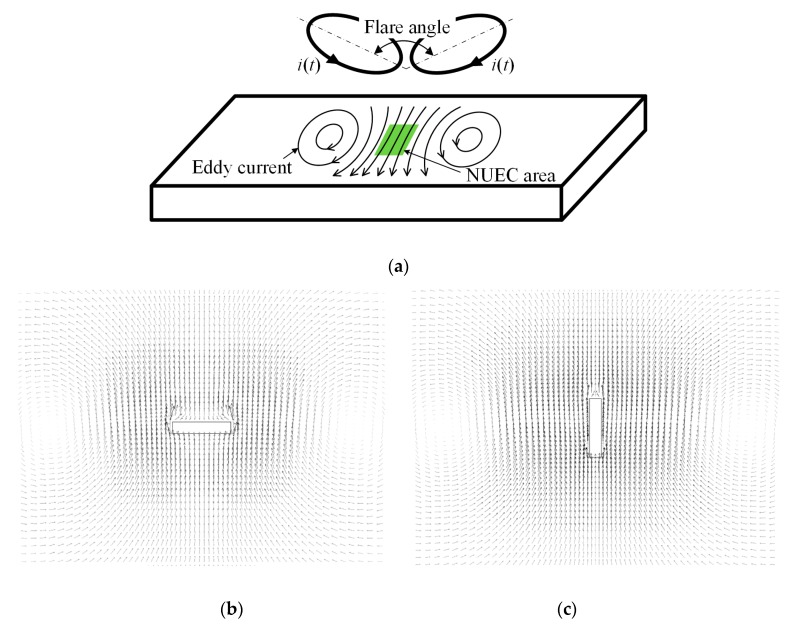
(**a**) Schematic of dual-coil focusing probe with induced near-unidirectional eddy current (NUEC) in the specimen and (**b**,**c**) eddy current interaction with a defect aligned in a perpendicular and parallel direction, respectively.

The focusing probe, as depicted in Figure 1a, typically consists of a pair of oppositely connected coils [16,17]. The excitation current in the two coils are of the same amplitude but in an opposite direction. In the junction zone, coil currents are approximately in the same direction. If the excitation current is alternating, then the eddy current induced in the conductor will form a focused and near-unidirectional eddy current (NUEC) area under the junction zone, shown by the green-colored area in the figure. When a defect is present in this region, the linear eddy current path will be distorted because electric current always tends to flow along a path with the least resistance. Figure 1b sketches the distribution of eddy current vectors around a defect. It can be seen that the original unidirectional eddy current makes a detour around the defect, which thus generates a secondary magnetic field normal to the specimen surface. If a magnetic sensor is applied in the vicinity, then the defect information can be obtained.

The induced eddy current in the conductor is the sum of the eddy currents induced by the two coils. Due to the antisymmetric excitation, the directions of eddy current induced magnetic flux at two sides of the central region are opposite in polarities. When an induction coil is used as the detection sensor, the electromotive forces generated by magnetic flux of the two sides will cancel each other out, rendering a zero output even if the coil is above the defect center [25]. Furthermore, the coil’s large footprint will lead to a low spatial resolution for the detection of a small defect. In this case, a small magnetic sensor might be more preferable.

The flare angle of the focusing coil affects the focusing field’s distribution and density. It was found that for a single-turn focusing coil, as the flare angle decreases, the maximum value of eddy current density increases while the field uniformity decreases sharply [17]. However, multi-turn coils with larger inductance are more practical and common in ECT applications and thus, the effect of flare angle on the focusing effect needs to be considered further.

### 2.2. Rotating focused EC field

The strength of the normal magnetic field that carries the defect information is related to the level of the eddy current–defect interaction. The more severe the perturbation caused by the defect is, the stronger the resultant magnetic field will be. Let us consider the scenarios when the defect lies in different directions with respect to the EC flow. It is obvious that the perturbation will be the strongest when the defect is perpendicular to eddy currents, as will be the normal magnetic field, which is the case shown in Figure 1b. When the defect is parallel to eddy currents (see Figure 1c), the perturbation will be the smallest, leading to a much weaker magnetic field, which might be difficult to identify from the receive signal with strong ambient noises. Therefore, it is necessary to improve the focusing probe for actual nondestructive testing (NDT) applications, where the defect orientation is random in the test object.

**Figure 2:**
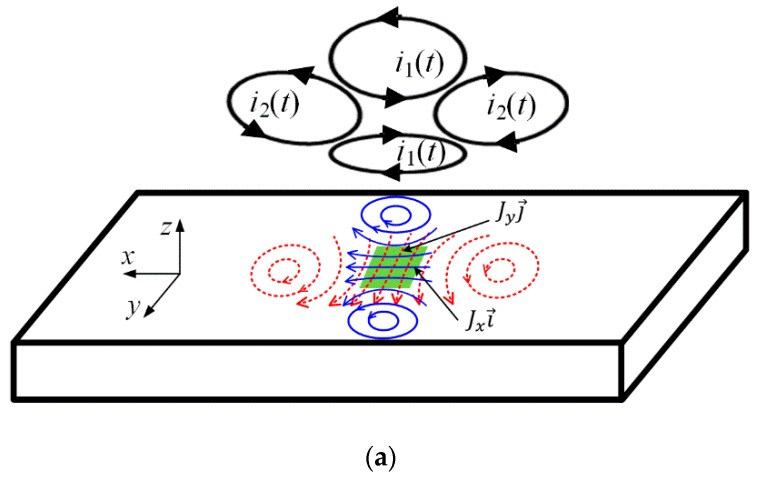

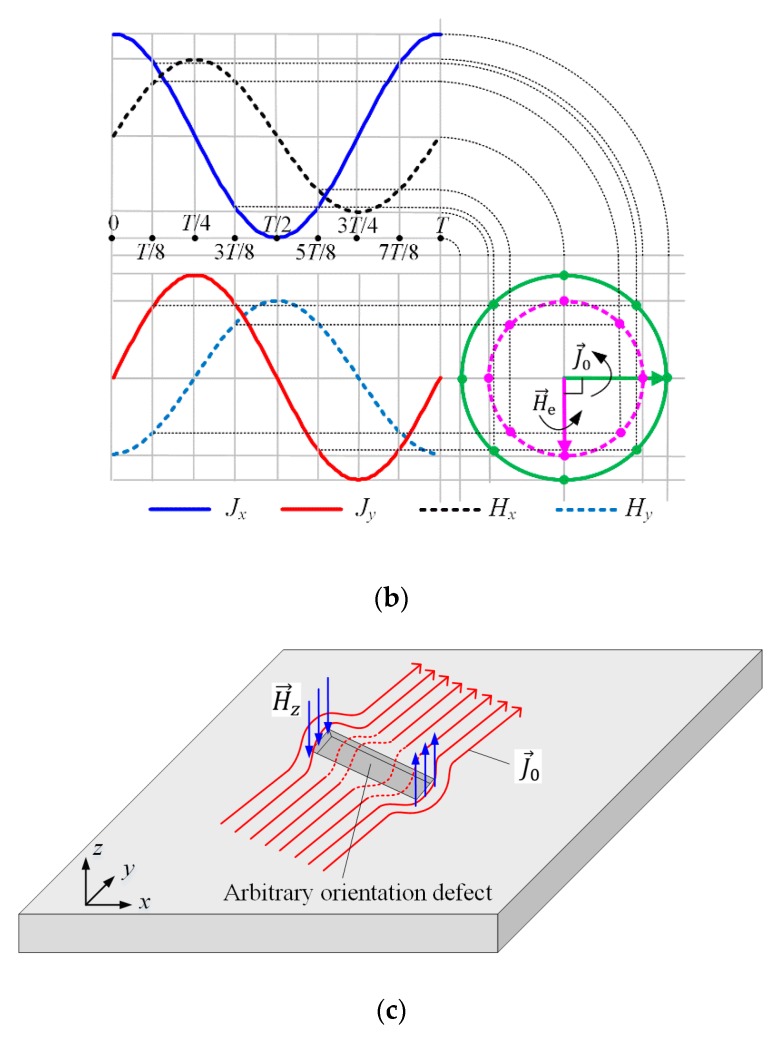
(**a**) Schematic of the rotating focused field eddy current testing (ECT)probe, (**b**) mathematical description of the generation of a rotating field and (**c**) illustration of the *z*-direction magnetic field generated by the eddy current detoured at the defect ends.

Inspired by the idea of rotating current excitation, a rotating focused field ECT probe is presented. It consists of two pairs of focusing sub-probes placed orthogonally to each other, as shown in Figure 2a. The two coils of one sub-probe carry a harmonic current, while the other sub-probe coils carry another current with the same amplitude as the former but with a 90 degrees phase shift. Near-unidirectional eddy currents $J_{x}\vec{i}$ and $J_{y}\vec{j}$ are then generated in the specimen area just under the probe center, as marked by green on the specimen surface. In this specific area with two orthogonally flowing linear eddy currents, the resultant eddy current field ${\vec{J}}_{0}$ can be written as [22]:

(1) $${\vec{J}}_{0}=J_{x}\vec{i}+J_{y}\vec{j}$$

where $J_{y}=\left|J_{x}\right|∠\left(\varphi_{x}+90^{{}^{\circ}}\right)$ with $\varphi_{x}$ denoting the phase of $J_{x}\;$ and $\vec{i}$ and $\vec{j}$ are unit vectors along the *x*-axis and *y*-axis, respectively.

The eddy current field ${\vec{J}}_{0}$ will induce a secondary magnetic field ${\vec{H}}_{e}$ and the relation between them is expressed as Maxwell–Ampere’s law:

(2) $$\nabla \times {\vec{H}}_{e}={\vec{J}}_{0}.$$

In the present case, the induced ${\vec{H}}_{e}$ has the $H_{x}$ and $H_{y}$ components, which are respectively generated by the $J_{y}$ and $J_{x}$ components, so equation (2) can be written as:

(3) $$-\frac{\partial H_{y}}{\partial z}\vec{i}+\frac{\partial H_{x}}{\partial z}\vec{j}=J_{x}\vec{i}+J_{y}\vec{j}.$$

According to the principle of vector superposition, it is inferable that the resultant vectors ${\vec{J}}_{0}\;$ and ${\vec{H}}_{e}$ are both rotating in the *xy* plane because their *x* and *y* components both have a 90-degree phase difference. They together form a rotating electromagnetic field at the specimen surface. This mechanism can be mathematically interpreted as follows—assuming $J_{x}$ is a cosine current $J_{0}\cos\left(\omega_{0}t\right)$ and thus $J_{y}$ is the sine current $J_{0}\sin\left(\omega_{0}t\right)$, then according to Euler’s formula, the synthetic current $J_{0}e^{j\omega_{0}t}$ forms a circle loci with radius equal to $J_{0}$. The same formation pattern holds for the magnetic field ${\vec{H}}_{e}$ and its radius vector is always orthogonal to that of ${\vec{J}}_{0}$, as described in Figure 2b. Since a circle is symmetrical under all rotations about its center, then in one cycle $T=2\pi /\omega_{0}$, there will always be EC vectors that align perpendicularly to an underlying defect, no matter in what orientation the defect is. Consequently, the induced current and field have the same sensitivity to arbitrary orientation defects at the specimen surface.

As indicated above, ${\vec{J}}_{0}$ and ${\vec{H}}_{e}$ are also mostly directed in the *xy* plane. When no defect occurs in the rotating field, there will be no magnetic field generated in the normal direction *z*. However, in the presence of defects, the unidirectional eddy current will have to detour around the defect and partly curl at the defect ends, yielding a non-zero *z*-direction magnetic field ${\vec{H}}_{z}$, as illustrated in Figure 2c. In this case, it is also worth mentioning that the moduli of $J_{x}$ and $J_{y}$ in Equation (1) will no longer be equal due to the defect-caused perturbation on the rotating field.

## 3. Model-based Study of Defect Detection

In this section, 3D finite element analysis (FEA) is performed by using ANSYS Workbench software to study the effectiveness of the presented probe for detecting arbitrary orientation defects. The unidirectional focusing probe is also studied for giving comparative results. The Solid236 element with 20 nodes is selected to model all the entities including the probe, the specimen and the air region. It is capable of modeling electromagnetic fields based on the edge-based magnetic vector potential method. In this method, the magnetic vector potential $\vec{A}$ and electric scalar potential V are used to represent the electromagnetic field. The basic equations involved are expressed as Maxwell–Faraday’s law, Maxwell–Ampere’s law, constitutive relations and potential functions:

(4) $$\nabla \times \vec{E}=-\frac{\partial \vec{B}}{\partial t}$$

(5) $$\nabla \times \vec{H}=\vec{J}+\varepsilon \frac{\partial \vec{E}}{\partial t}={\vec{J}}_{s}+{\vec{J}}_{e}+{\vec{J}}_{v}+\varepsilon \frac{\partial \vec{E}}{\partial t}$$

(6) $$\vec{B}=\mu \vec{H}$$

(7) $$\vec{J}=\sigma \vec{E}$$

(8) $$\vec{B}=\nabla \times \vec{A}$$

(9) ∇×∇V≡0,

where the various quantities are defined as

$\vec{E}$: Electric field intensity (V/m)

$\vec{B}$: Magnetic flux density (T)

$\vec{H}$: Magnetic field intensity (A/m)

$\vec{J}$: Total conduction current density (A/m^2^)

${\vec{J}}_{s}$: Applied source current density (A/m^2^)

${\vec{J}}_{e}$: Induced eddy current density (A/m^2^)

${\vec{J}}_{v}$: Velocity current density (A/m^2^)

t: Time (s)

ε: Permittivity (F/m)

μ: Magnetic permeability (H/m)

σ: Conductivity (S/m)

Substituting Equation (8) into Equation (4) results in the following equation:

(10) $$\nabla \times \left(\vec{E}+\frac{\partial \vec{A}}{\partial t}\right)=0.$$

Invoking Equation (9), $\vec{E}$ can be written as

(11) $$\vec{E}=-\frac{\partial \vec{A}}{\partial t}-\nabla V.$$

Finally, by substituting Equations (11), (6) and (7) into Equation (5), the governing equations are obtained:

(12) $$\nabla \times \frac{1}{\mu }\nabla \times \vec{A}+\sigma \left(\frac{\partial \vec{A}}{\partial t}+\nabla V\right)+\varepsilon \left(\frac{\partial^{2}\vec{A}}{\partial t^{2}}+\nabla \frac{\partial V}{\partial t}\right)=0\;\mathrm{in}\;\mathrm{the}\;\mathrm{conducting}\;\mathrm{region},$$

(13) $$\nabla \times \frac{1}{\mu }\nabla \times \vec{A}={\vec{J}}_{s}\;\mathrm{in}\;\mathrm{the}\;\mathrm{free}\;\mathrm{space}\;\mathrm{region}.$$

**Figure 3:**
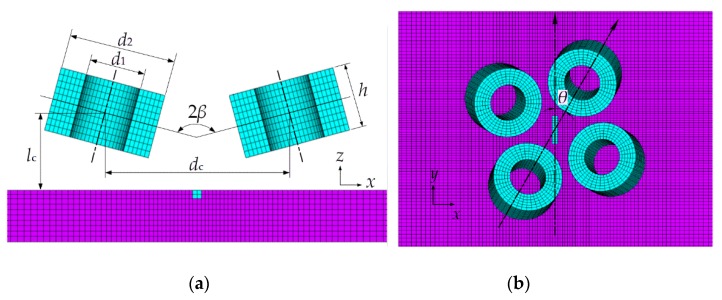
Finite element analysis (FEA) model of the presented probe. (**a**) Side view and (**b**) top view. In (**a**) only one sub-probe (two coils) are plotted to clearly describe the geometry parameters.

Figure 3a,b depict the FEA model. For clarity, the elements of air properties are not plotted except those inside the defect. The geometry parameters are defined here—2*β*, the flare angle between two coils of the sub-probe; *l*_c_, the distance from the coil center to the specimen surface; *d*_c_, the center distance between two coils; *θ*, the angle from the symmetric line of one sub-probe to the defect lengthwise direction.

The specimen under test is a carbon steel plate with parameters listed in Table 1. A groove with the size of 10 mm × 2 mm × 2 mm (length, width, depth) is set on the central top surface of the specimen to simulate the defect. Table 2 lists the geometric parameters of the probe coil.

**Figure 4:**
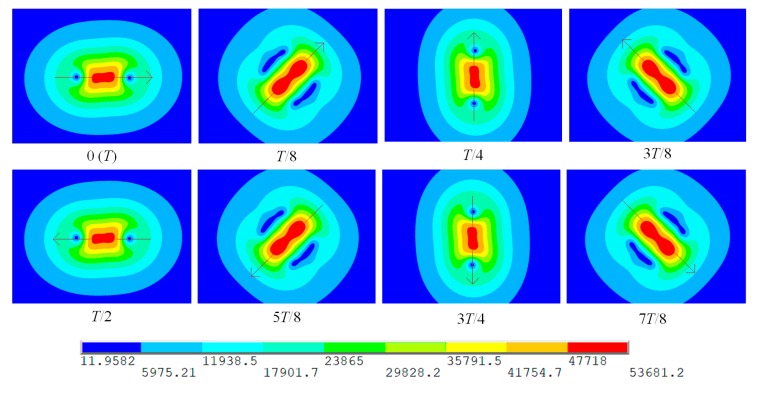
Distribution of the induced eddy current density on the surface of the specimen with the rotating field probe for different times of one excitation cycle (unit: A/m^2^).

Figure 4 shows the distribution of the induced eddy current on the surface of the specimen with the rotating field probe. The eddy current under the probe center is oriented towards varying directions as the time changes. It is dynamically formed in a counterclockwise direction in a whole excitation cycle *T* and indeed exhibits a spatial rotating feature. This observation is consistent with the prediction described in Figure 2b and therefore proves the effectiveness of the rotating field probe.

**Figure 5:**
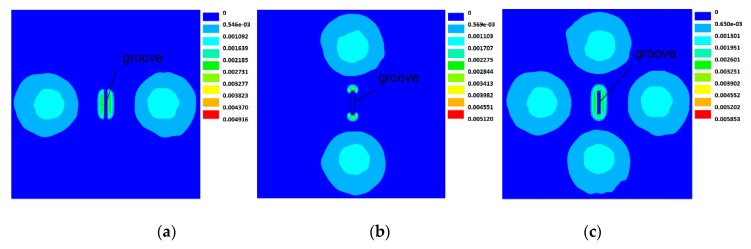
Simulated normal magnetic flux density distribution at the specimen surface with an identical groove interacting (**a**) in parallel with and (**b**) perpendicular to the flowing unidirectional eddy current and (**c**) the rotating field eddy current, respectively (unit: T).

Figure 5 compares the distribution of normal magnetic flux density on the specimen surface with the groove lain in eddy currents induced by the focusing probe and the rotating field probe. Figure 5a and b respectively show the cases with eddy current flow parallel and perpendicular to the groove length direction. It can be seen that the normal magnetic field only arises at the two edges along the current flow. In contrast, in the rotating field as shown in Figure 5c, the normal magnetic field is produced alongside all four edges and with uniform density distribution, which provides omni-directional information about the groove and therefore makes the groove able to be sensed from any orientation. Furthermore, by checking the color variation against the legend, the magnetic flux density is found to decrease sharply from the groove edge to its outer vicinity, which is attributed to the focusing effect and can contribute towards enhancing the defect-related signal and suppress the influence of the background field.

The simulated normal magnetic flux density ${\vec{B}}_{z}$ is a complex quantity and can be expressed in terms of the magnitude $\left|{\vec{B}}_{z}\right|$ and the phase $\varphi_{Bz}$ as follows:

(14) |B→z|=(Re(B→z))2+(Im(B→z))2

(15) φBz=arg(B→z)=tan−1(Im(B→z)Re(B→z)),

where Re and Im are the real and imaginary part of ${\vec{B}}_{z}$, respectively.

### 3.1. Detection of Arbitrary Orientation Defects

To simulate different defect orientations in a FEA model, the probe is rotated step-by-step to change the value of angle *θ*. In this way, the meshes of the defect region can be kept exactly the same in different simulation models, thus ensuring the comparability of the simulation results. Considering the structure symmetry, a set of *θ* from 0 to 90 degrees by steps of 15 degrees is used for the focusing probe model. For rotating the focused field ECT probe, a set of *θ* from 0 to 165 degrees (*θ* = 180° is the same as the case of 0°) by steps of 15 degrees is used.

Simulation data is processed by three steps to obtain the signal. First, a 30 mm long data path along the defect length direction is aligned, with 2.5 mm distance off the plate and middle point above the defect center. Data sets representing the normal magnetic flux density ${\vec{B}}_{z}$ mapped to this path are extracted. Afterwards, all the data are subtracted by a data set acquired from a defect-free model, yielding a differential signal with the notation diff. $\;B_{z}$. Finally, the magnitude and phase angle of diff. $B_{z}$ are calculated by using formulas (14) and (15).

**Figure 6:**
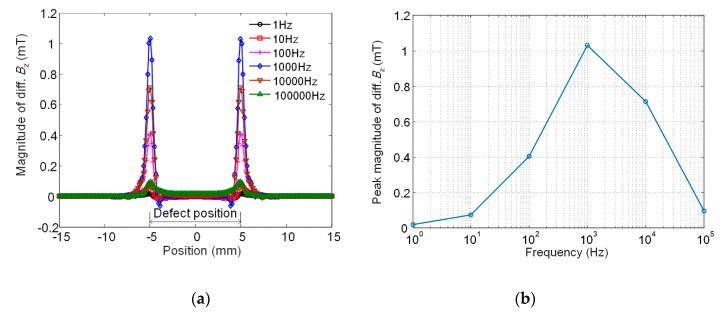
Simulated results by using the rotating probe excited by harmonic currents with different frequencies. (**a**) Signal magnitude versus position and (**b**) plot of peak magnitude with excitation frequency.

The excitation frequency is a key parameter in eddy current testing. For a given conductive material, the frequency is not only concerned with the skin depth but also with the probe signal magnitude. An optimal frequency exists at which the maximum signal is retrieved for a fixed-size defect [27]. Figure 6 gives the simulated results by using the rotating probe excited by harmonic currents with the same density of 1.67 × 10^6^ A/m^2^ but different frequencies. As seen in Figure 6a, the magnitude signal curve exhibits two symmetric peaks whose positions correspond to the two lengthwise ends of the defect. This phenomenon agrees with the fact that the defect edge causes a severe distortion to eddy current flow and thus triggers a significant normal magnetic field. Figure 6b shows that the probe signal magnitude reaches the maximum at the frequency of 1 kHz as the frequency varies from 1 Hz to 100 kHz. Considering the need for detecting a deeper defect, a compromise is made and the 100 Hz is selected as the excitation frequency.

**Figure 7:**
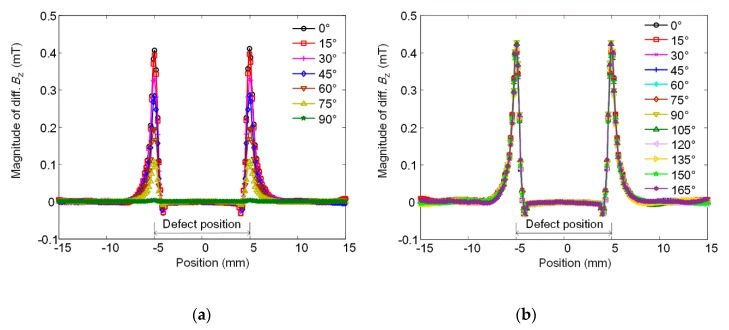
Plot of magnitude of simulated signals against position for defects with different orientations (varying *θ*). (**a**) Focusing probe; (**b**) rotating focused field probe.

The simulated signal magnitudes of the focusing and the rotating probes for detecting defects with various orientations are shown in Figure 7a,b, respectively. As the defect orientation varies (i.e., *θ* increases), the peak value of the focusing probe signal decreases significantly, while that of the rotating focused field probe remains almost unchanged. From this comparison, it can be concluded that the strategy of integrating the focusing coil into the rotating field probe works well and the hybrid probe shows uniform sensitivity to arbitrary orientation defects in the specimen.

**Figure 8:**
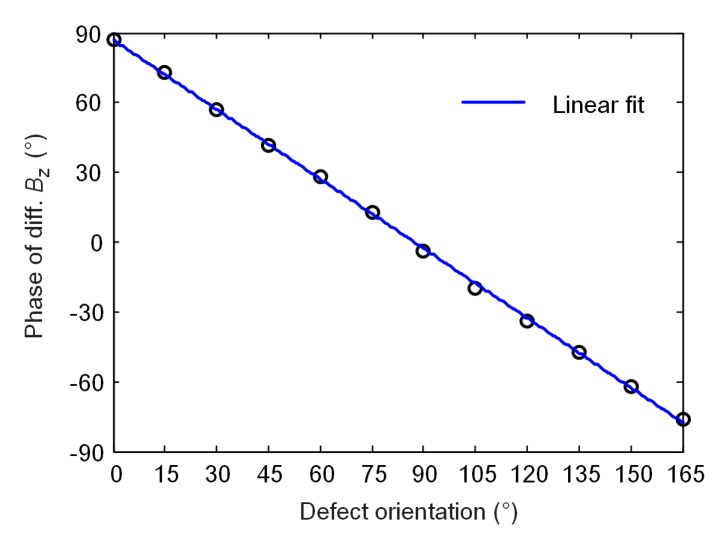
Variation of the phase angle of the simulated signal with defect orientation by using the rotating focused field probe.

Further, the signal data with maximum peaks, arising at the defect edge as analyzed above, is extracted and its phase angles are calculated. Figure 8 shows the variation of phase angle with the defect orientation for using the rotating probe. The fitted line indicates a descending linear relationship between them, which suggests a potential method for determining the defect orientation.

### 3.2. Effect of Flare Angle

**Figure 9:**
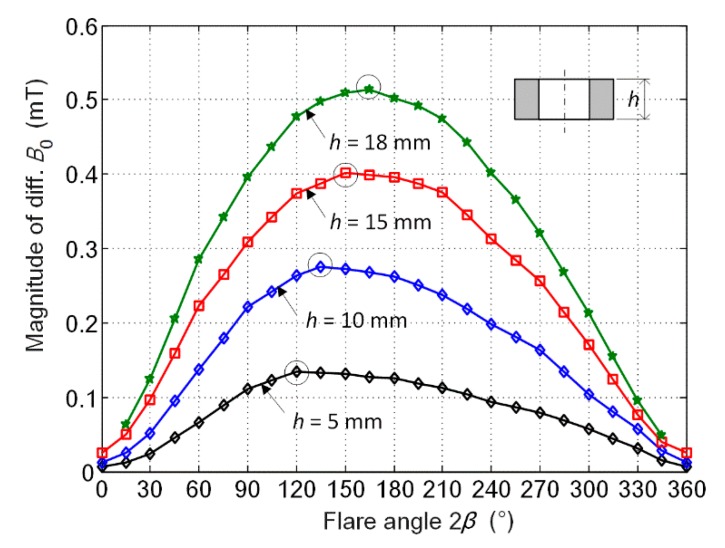
Variation of simulated signal magnitude with probe’s flare angle for probe coil height *h* = 18, 15, 10 and 5 mm, respectively.

As an important factor, the effect of the flare angle 2*β* on the performance of the rotating focused field probe is studied. Figure 9 shows the simulation result for 2*β* varying from 0 to 360 degrees by steps of 15 degrees. Note the magnitude represents the curve’s peak magnitude such as that plotted in Figure 7. It can be seen that the signal magnitude slowly increases to reach its maximum and afterwards decreases until the end. For the given probe, the peak value appears at 2*β* = 150°. This peak point is not fixed but depends on the coil geometry. As the coil height *h* decreases from 18 mm to 5 mm, the flare angle corresponding to the peak point (circled on the curve) shows a gradual decrease as well. The curve of *h* = 18 mm does not have values at 2*β* = 0° and 360° because in either case the adjacent coils of the probe will interfere with each other. When the coil height increases, the curves will be more incomplete.

**Figure 10:**
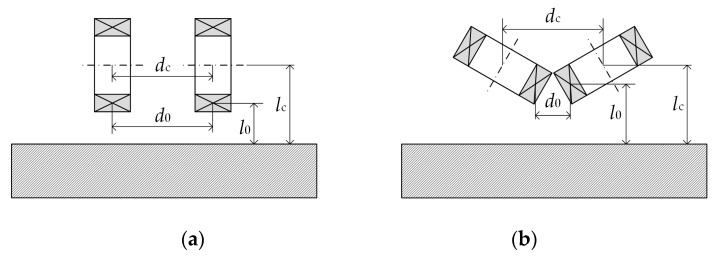

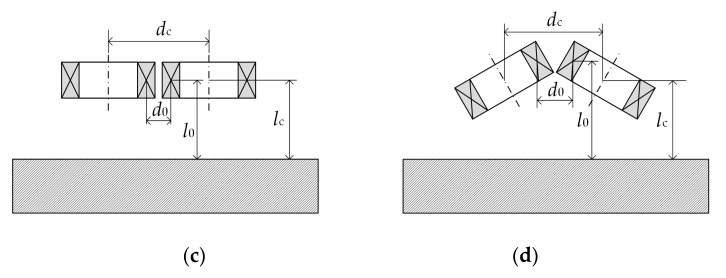
Illustration of the rotating probe’s two opposite coils with the flare angle equal to (**a**) 0°, (**b**) 120°, (**c**) 180° and (**d**) 240°, respectively. Note *d*_c_ and *l*_c_ are fixed values.

It has to be pointed out that the change rule is presented based on the given probe geometry with fixed coil center distance *d*_c_ and fixed coil center lift-off *l*_c_ as shown in Figure 3a. When the flare angle changes, the distance from the bottom of the coil to the specimen also changes. For a single tilted coil, the eddy current is stronger when the coil is closer to the specimen [28]. For the presented probe with four tilted coils, the concerned rotating field is the superposition of the field generated by four coils. To accommodate adjacent coils, an adequate space between each pair of oppositely tilted coils is required. In this case, the eddy current density is related not only to the distance from the coil to the specimen but also to the space between opposite coils. Since eddy current produced by a single tilted coil tends to focus underneath the near side of the coil [28], two focusing coils with a smaller space between their inner sides will result in a stronger net eddy current field. However, when the flare angle changes, the distance from the coil on the near side of the specimen (denoted by *l*_0_) and the space between opposite coils (denoted by *d*_0_) will change in opposite directions, as described by three typical cases in Figure 10 when the flare angle is 0°, 120° and 180°, respectively. When the flare angle is 0°, *l*_0_ is the minimum value and *d*_0_ is the maximum. The opposite situation occurs when the flare angle changes to 180°. Then, the intermediate case as shown in Figure 10b has the potential to generate stronger eddy current underneath the probe center. When the flare angle exceeds 180°, the inner side of the coils comes up while the outside goes down, as is the case with a flare angle of 240°, shown in Figure 10d. Taking the case with a 180° flare angle as the reference, due to the overall higher values of *l*_0_, eddy current generated by the probes with flare angles greater than 180° will be weaker than eddy current generated by the probes with rival flare angles, for instance, the case with a 240° flare angle versus the case with a 120° flare angle. This explains the curves in Figure 9 with the peak values emerging before 180° and with steeper left rising parts than right descending parts.

## 4. Experimental Verification

**Figure 11:**
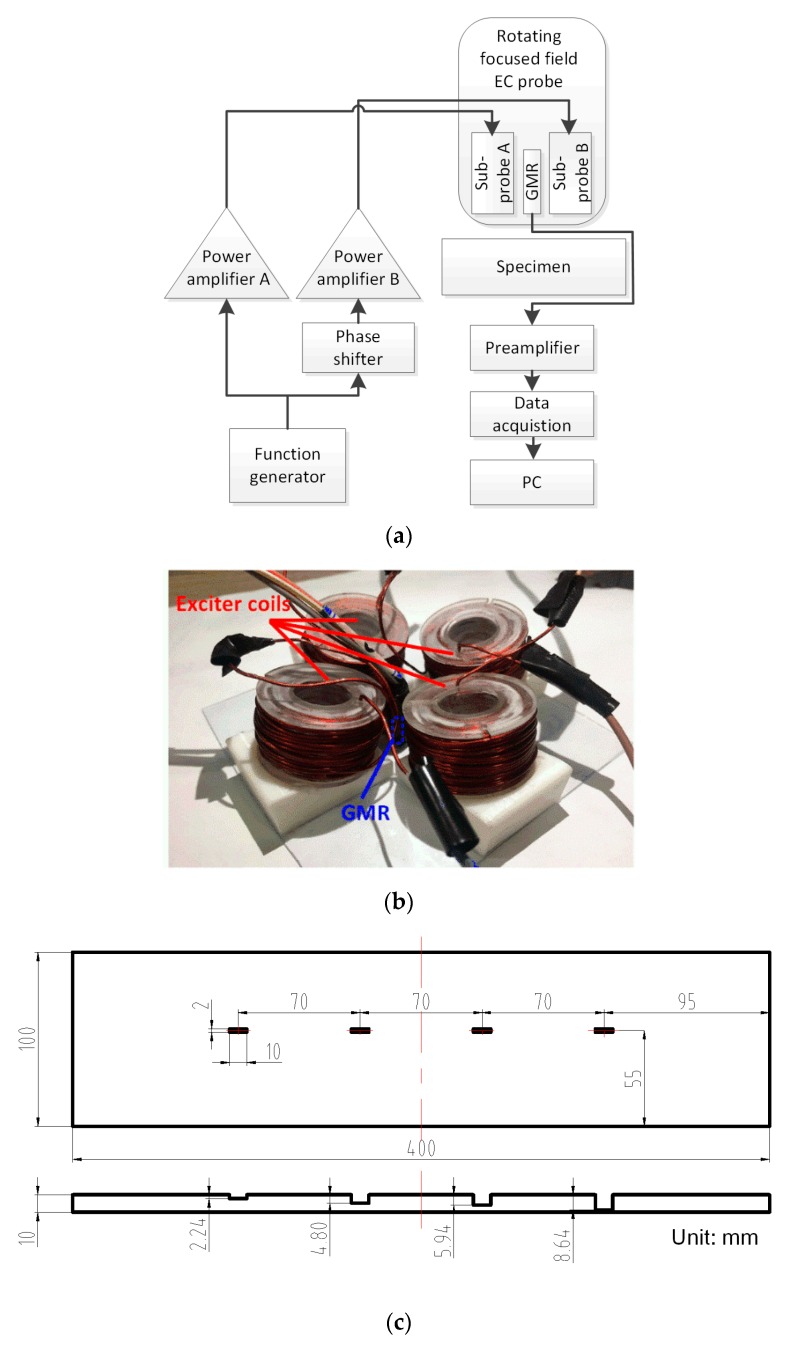
Experiment system setup. (**a**) Composition diagram, (**b**) probe and (**c**) specimen.

Experimental study was also conducted to verify the probe performance. Figure 11 shows the composition, prototype probe and the specimen of the experiment system. The probe has a flare angle of 150 degrees and its coil parameters are the same as those given in Table 2. The specimen is a Q235 steel plate with four prefabricated grooves, with the same length and width but different depths, as shown in Figure 11c. Due to the machining error, the actual depths of the grooves are 2.24 mm, 4.80 mm, 5.94 mm and 8.64 mm, measured by a Vernier caliper with an accuracy of 0.02 mm, which deviate respectively from their design values of 2 mm, 4 mm, 6 mm and 8 mm. A sine wave of 100 Hz frequency and 0° initial phase is generated by a function generator and output through two channels. One is directly amplified by the power amplifier A, while the other is sent to the phase shifter module to form a 90-degree phase shift before being amplified by the power amplifier B. After that, the two amplified sinusoidal currents are fed into the sub-probe A and B respectively. The two power amplifiers are of the same type and with identical performances.

Considering the low excitation frequency, a GMR AA002-02 sensor from NVE Corporation [29] was used as the detection sensor. It has a linear range from 1.5 to 10.5 Oe (1 Oe = 0.1 mT in air) and presents the highest sensitivity of [3.0, 4.2] mV/V-Oe in NVE AA00x series [29]. The GMR sensor was mounted in the probe center surrounded by the excitation coils with its sensitivity axis along the normal direction of the specimen surface. This compact structure helps decrease the probe design lift-off. A small circuit board for mounting the GMR was used. Since the GMR sensor operates in a unipolar mode, a small permanent magnet is placed near the sensor to bias the sensor in the middle of the linear range [30]. Taking all factors into account, the total lift-off of the GMR sensor is around 2.5 mm. The GMR output is conditioned by a custom-made preamplifier and then digitalized by a NI data acquisition system linked with a personal computer for subsequent signal processing. The magnitude and phase angle are extracted by using a digital lock-in amplifier program coded in LabVIEW.

**Figure 12:**
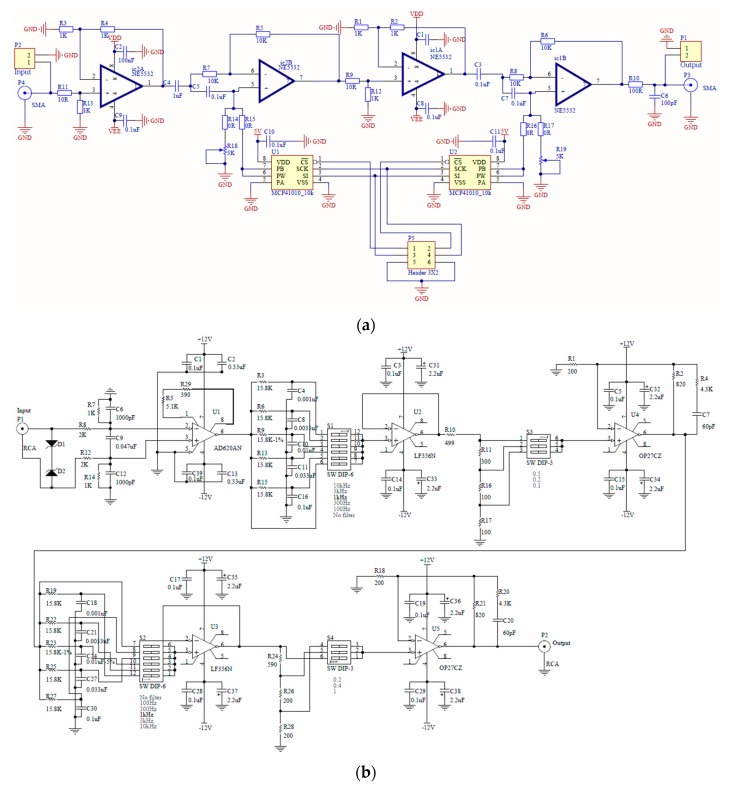
Schematic circuit diagrams of the homemade (**a**) phase shifter and (**b**) preamplifier.

The excitation current after the power amplifier has an amplitude of 1 A, which matches the corresponding current density used in the simulation. The phase shifter module is homemade and can generate 0 to 360 degrees shift at the frequency range of a few hertz up to 1 kHz. It employs a two-stage shifter structure and provides two multi-turn trimpots with high resolution for manual adjustment to obtain a high accuracy of phase shift. The custom-made preamplifier functions as a signal filter and an amplifier whose cut-off frequency and gain factor can be configured as needed. During the experiment, the cut-off frequency and gain factor were set as 1 kHz and 10, respectively. Figure 12 presents the schematic circuit diagrams of the phase shifter and the preamplifier. Note that R18 and R19 in Figure 12a represent the trimpots, while S1 to S4 in Figure 12b denote the DIP switches for setting cut-off frequency and gain factor.

**Figure 13:**
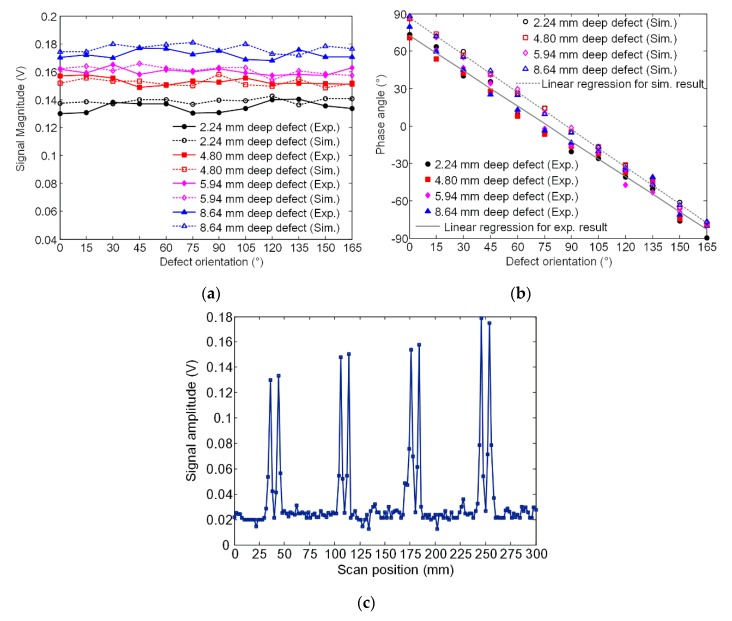
Comparison of experiment and simulation results by in-situ rotating the presented probe above the defect: (**a**) signal magnitude and (**b**) phase angle. (**c**) Experimental signal amplitude obtained by scanning the probe along the lengthwise center line of four defects.

First, the probe was placed with the GMR sensor right above the width edge of the defect. In order to simulate the variation of defect orientation, the probe was in situ rotated step by step on the specimen surface. Figure 13a and b show the experiment signal magnitude and phase angle versus the defect orientation for four different depth defects. For comparison, simulation results are also shown. The conversion of simulation data from mT to V are based on the following relationship—the response curve of GMR AA002-02 at room temperature is tested for 100 Hz applied magnetic field and its sensitivity in the linear range is determined as 3.2 mV/V-Oe. The gain factor of the preamplifier is 10 and thus, the equivalent voltage is 0.32 V per 1 mT. It can be seen that the experiment results are basically in agreement with the simulation results—as the defect orientation varies, the magnitude holds stable while the phase angle exhibits a linear variation. The observed slight deviations between experiment and simulation results are tolerable considering various kinds of influence factors including the linear and homogeneous material assumption made in the simulation, the GMR sensor calibration error and the phase shifting caused by the analog electronic devices involved in experiments. Furthermore, according to Figure 13a, as the defect depth increases, the signal magnitude increases as well, while the signal phase shows no significant change. All the above findings indicate that the signal magnitude can be used to evaluate the defect depth, regardless of the defect orientation.

A line scan using the presented probe is also carried out on the specimen. The scan path is along the lengthwise center line of four defects. To avoid the edge effect, the beginning and ending points are 50 mm away from the edges, so the total scanning distance is 300 mm. All scan points are operated manually with a step of 2 mm. Figure 13c shows the scanning results. From left to right, the four amplitude-rising signals correspond well with the four defects with depth of 2.24, 4.80, 5.94 and 8.64 mm, respectively. Good SNR was also observed. Small signal fluctuations are probably due to the inaccurate manual operation and interferences caused by the intrinsic non-uniform magnetic permeability of the carbon steel.

## 5. Conclusions

A novel rotating focused field eddy-current probe for the detection of arbitrary orientation defects is presented. The magnetic field focusing method of the figure-8-shaped coil is integrated into the generation of the rotating excitation field. The hybrid excitation strategy gives the designed probe advantages in both structure and function. Instead of a stacked structure, the probe employs a symmetric and equal lift-off configuration for all excitation coils and the receiving GMR sensor, thus making a small design lift-off and avoiding the additional excitation adjustment encountered in existing rotating field probes. By optimizing the probe’s flare angle, the probe shows the capability of focusing eddy current at the defect area while weakening it at the background area, which contributes to a defect signal with a high signal-to-noise ratio. Feasibility of the probe to detect arbitrarily oriented defects was studied using simulation and validated by experiment tests on a carbon steel plate. A line scan over defects with different depth was also performed to demonstrate the probe’s focusing effect.

It should be noted that, in this paper, only the effect of the flare angle was preliminarily analyzed for the fixed-coil-geometry probe. Other parameters including the coil shape, coil dimensions, the distance between each pair’s coils are factors related to the probe’s performance. Multi-parameter optimization is pending in future work. In addition, more work will be done to further validate and refine the probe performances, including developing a C-scan presentation to image the defect and performing more experiments to study the detection accuracy for fine cracks and deep buried defects.

## Figures and Tables

**Table 1:** Specimen parameters.

Parameter	Value
Length/mm	380
Width/mm	380
Thickness/mm	10
Conductivity/S·m^−1^	5 × 10^6^
Relative permeability	329.5

**Table 2:** Coil parameters.

Parameter	Value
Inner diameter *d*_1_/mm	13
Outer diameter *d*_2_/mm	25
Height *h*/mm	15
Number of turns	150
Wire diameter/mm	0.8
Center distance *d*_c_/mm	43
Center lift-off *l*_c_/mm	18
